# Supplementary material for: Performance of a deep learning based neural network in the selection of human blastocysts for implantation
Source: eLife. 2020 Sep 15;9:e55301. doi: 10.7554/eLife.55301 (PMC7527234; doi:10.7554/eLife.55301)
Supplement: Supplementary file 1. — (A) Patient population characteristics. All embryo images (except the PGT screened embryos) utilized for experiments reported in the study were obtained from cycles that belong to the presented distribution of parameters. All values in table are presented as median along with the range unless noted otherwise. (B) Total number of transfer outcomes for embryos selected by the network. A total of 102 fresh-transfer embryos had known implantation outcomes (45 embryos implanted). Twenty-eight frozen transfers were performed by the clinic where 18 implanted. The table lists only embryos which were selected by the network with known outcomes for both fresh cycles and in frozen subsequent transfers. (C) Cycle characteristics of the euploid test set. Embryos used in the euploid embryo differentiation experiment based on the implantation outcomes, originated from cycles that belong to presented distribution of characteristics. These cycles are independent of the original 97 patient cohort test set and also the training data sets. All values in table are presented as median along with the range unless noted otherwise. [file elife-55301-supp1.docx]

Title: Performance of a deep learning based neural network in the selection of human blastocysts for implantation

**Authors:** Charles L Bormann^1,2^†, Manoj Kumar Kanakasabapathy^3^†, Prudhvi Thirumalaraju^3^†, Raghav Gupta^3^, Rohan Pooniwala^3^, Hemanth Kandula^3^, Eduardo Hariton^1^, Irene Souter^1,2^, Irene Dimitriadis^1,2^, Leslie B. Ramirez^4^, Carol L. Curchoe^5,6^, Jason E. Swain^6^, Lynn M. Boehnlein^7^, Hadi Shafiee^2,3^*

**Affiliations:**

^1^Division of Reproductive Endocrinology and Infertility, Department of Obstetrics and Gynecology, Massachusetts General Hospital, Harvard Medical School, Boston, MA, USA

^2^Harvard Medical School, Boston, MA, USA.

^3^Division of Engineering in Medicine, Department of Medicine, Brigham and Women’s Hospital, Harvard Medical School, Boston, MA, USA.

^4^Extend Fertility, New York, NY, USA.

^5^San Diego Fertility Center, San Diego, CA, USA.

^6^Colorado Center for Reproductive Medicine IVF Laboratory Network, Englewood, CO

^7^Division of Reproductive Endocrinology and Infertility, Department of Obstetrics and Gynecology, University of Wisconsin, Madison, WI, USA.

*Corresponding author. E-mail: hshafiee@bwh.harvard.edu

† These authors contributed equally to this work

**Supplementary file 1A.** **Patient population characteristics.** All embryo images (except the PGT screened embryos) utilized for experiments reported in the study were obtained from cycles that belong to the presented distribution of parameters. All values in table are presented as median along with the range unless noted otherwise.

| Characteristics information |  |
| --- | --- |
| Cycle characteristics | Test data set (n=97) |
| Age, years | 34 (43 - 24) |
| Day 3 follicle stimulating hormone, IU/L | 7 (12 - 4) |
| ^a^Body mass index, kg/m^2^ | 23 (39 - 17) |
| Oocytes retrieved, n | 12 (27 - 6) |
| Mature (MII) oocytes, n | 11 (27 - 4) |
| Normally fertilizations (2PN), n | 8 (20 - 3) |
| ^b^Fertilization method, n (%) |  |
| Conventional insemination | 27 (27.84) |
| Intracytoplasmic sperm injection | 70 (72.16) |
| ^b^Infertility Diagnosis, n (%) |  |
| Diminished ovarian reserve | 7 (7.22) |
| Polycystic ovary syndrome | 11 (11.34) |
| Uterine | 3 (3.09) |
| Unexplained | 15 (15.46) |
| Male factor | 37 (38.14) |
| Tubal | 10 (10.31) |
| Endometriosis | 1 (1.03) |
| Idiopathic | 1 (1.03) |
| Other | 12 (12.37) |
| ^a^ n = 91  ^b^ Values represent the absolute count and percentage within the test set | |

**Supplementary file 1B.** **Total number of transfer outcomes for embryos selected by the network.** A total of 102 fresh-transfer embryos had known implantation outcomes (45 embryos implanted). 28 frozen transfers were performed by the clinic where 18 implanted. The table lists only embryos which were selected by the network with known outcomes for both fresh cycles and in frozen subsequent transfers.

| **Embryos selected by the network with known outcomes** |  |  | |
| --- | --- | --- | --- |
|  | **113 hpi** | |  |
|  |  | |  |
| **Fresh transfers** | 44 | |  |
| **Fresh transfers with successful implantation** | 26 | |  |
| **Frozen transfers** | 5 | |  |
| **Frozen transfers with successful implantation** | 4 | |  |

**Supplementary file 1C. Cycle characteristics of the euploid test set**. Embryos used in the euploid embryo differentiation experiment based on the implantation outcomes, originated from cycles that belong to presented distribution of characteristics. These cycles are independent of the original 97 patient cohort test set and also the training data sets. All values in table are presented as median along with the range unless noted otherwise.

| PGT screened euploid embryo cycle characteristics | |
| --- | --- |
| Cycle characteristic | **Test data set (n = 97)** |
| * Successful implantations, n (%) | 57 (58.76) |
| Available embryos, n | 5 (12, 1) |
| Patient age, n | 35 (47, 25) |
| Body mass index, kg/m^2^ | 23 (37, 17) |
| Day 3 follicle stimulating hormone, IU/L | 7 (13, 3) |
| Oocytes retrived, n | 15 (25, 2) |
| Normally fertilized (2PN) embryos, n | 9 (20, 2) |
| Number of blastocysts biopised, n | 5 (11, 1) |
| * Infertility diagnosis, n (%) |  |
| Diminished ovarian reserve | 12 (12.37) |
| PCOS | 6 (6.19) |
| Unexplained | 16 (16.49) |
| Male factor | 20 (20.62) |
| Tubal | 9 (9.28) |
| Other | 31 (31.96) |
| Recurrent pregnancy loss | 3 (3.09) |
| * Values represent the absolute count and percentage within the test set | |
